# Medical Reciprocity

**Published:** 1899-12

**Authors:** 


### MEDICAL RECIPROCITY.

At the annual meeting of the Massachusetts Surgical and Gynæcological Society held in Boston December 13th, 1899, the following resolutions, suggested by the president, Dr. J. P. Rand, in his annual address, were unanimously adopted:

WHEREAS, The Massachusetts Surgical and Gynæcological Society, believing that the laws for medical registration, as they appear in many States, are unjust to the reputable practitioner who for any reason may desire to change his location from one State to another; therefore be it

*Resolved,* That this society call upon the American Institute of Homœopathy, as the oldest national medical organization in this country, to take some action towards bringing about a uniform system for registration in medicine, whereby a physician legally qualified to practice in any State or territory of this Union, or in the District of Columbia, may be allowed to register for practice in any other State or territory of this Union or in the District of Columbia upon the presentation of a verified certificate and the payment of a nominal fee.

*Resolved,* That a copy of these resolutions be forwarded to the chairman of the Legislative Committee, of the American Institute of Homœopathy, for such consideration as may be deemed expedient.

